# Pyroptosis: A Common Feature of Immune Cells of Haemodialysis Patients

**DOI:** 10.3390/toxins13120839

**Published:** 2021-11-25

**Authors:** Christof Ulrich, Leonie Kneser, Roman Fiedler, Julia Beckert, Susann Wildgrube, Eric Seibert, Sylvia Fick, Christoph Schäfer, Silke Markau, Bogusz Trojanowicz, Matthias Girndt

**Affiliations:** 1 Department of Internal Medicine II, Martin Luther University Halle-Wittenberg, 06120 Halle, Germany; roman.fiedler@uk-halle.de (R.F.); jule.beckert@googlemail.com (J.B.); info@nephrologie-vs.de (E.S.); sylvia.fick@uk-halle.de (S.F.); christoph.schaefer@uk-halle.de (C.S.); Silke.markau@uk-halle.de (S.M.); matthias.girndt@uk-halle.de (M.G.); 2 Agaplesion Ev. Klinikum Schaumburg, 57392 Oberkirchen, Germany; leonie@kneser.de; 3 BG Klinikum Bergmannstrost, 06120 Halle, Germany; susannwildgrube@gmx.de; 4 Nephrologisches Zentrum Villingen-Schwenningen, 78054 Villingen-Schwenningen, Germany; 5 Department of Visceral, Vascular and Endocrine Surgery, Martin Luther University Halle-Wittenberg, 06120 Halle, Germany; bogusz.trojanowicz@uk-halle.de

**Keywords:** haemodialysis, hypertension, pyroptosis, indoxyl sulfate, caspase-1, caspase-4, immune cells

## Abstract

NLRP-3 inflammasome activation can result in interleukin-1β (IL-1β) release and inflammatory cell death (pyroptosis). Caspase-1 is able to trigger both processes. However, other caspases, caspase-4, -5 and -8, are believed to initiate pyroptosis without affecting IL-1 secretion. In this study, we evaluated two cardiovascular risk groups, haemodialysis patients (HD) and patients with intact kidney function but high blood pressure (BP), to analyse the mechanisms driving pyroptosis. Twenty HD were age-, gender- and diabetes-matched to BP. We found a common pyroptotic pattern in both patient groups, at which pyroptosis rates but not IL-1 β levels were significantly higher in monocytes (HD vs. BP: *p* < 0.05), granulocytes (*p* < 0.01) and lymphocytes (*p* < 0.01) of HD patients. As uremic toxins are drivers of inflammation and regulated cell death, we applied a monocyte- and macrophage-like THP-1 model system to demonstrate that the protein-bound uremic toxin indoxyl sulfate (IS) is an inducer of pyroptotic cell death, particularly engaging caspase-4/caspase-5 and to a lesser extent caspase-8 and caspase-1. These data suggest that the uremic toxin IS can mediate pyroptosis in HD patients and the inflammatory caspase-4 and/or caspase-5 contribute to pyroptosis rates to a higher extent in comparison to caspase-1.

## 1. Introduction

To respond to pathogens and endogenous danger signals, cells of the innate immune system are endowed with the NLRP3 inflammasome. The formation of the active NLRP3 protein complex leads to caspase-1 activation, which in turn can result in cleavage not only of pro-IL-1β but also of gasdermin D. Active gasdermin D aggregates and forms pores in the cell membrane. The consequence of this is cell swelling and finally cell death, a process that is called pyroptosis. Thus, mature IL-1β can leave the cell via gasdermin-formed pores or after the complete lysis of cells. The eminent importance of IL-1β is supported by the fact that there are still other ways, i.e., microvesicles, lysosomes and exosomes, by which IL-1β can be transported and released [1,2,3]. Notably, pyroptosis is not necessarily linked to caspase-1 activation. Induced by cytosolic LPS, caspase-4 and -5 are also able to cleave gasdermin D [4,5]. Further on, activated caspase-8 can possibly not only induce apoptosis but also pyroptosis [6,7]. Therefore, it is quite possible that a pyroptotic event can occur without IL-1β being secreted.

Studying monocytic inflammasome activation in hypertensive patients with intact kidney function (BP) and haemodialysis patients (HD), we recently reported that though IL-1β was elevated in BP to a higher degree, HD had significantly higher monocytic pyroptosis rates. Extending this study, we firstly wanted to know if there is a common NLRP3 inflammasome activation profile in the immune cells of our patients: Do monocytes, granulocytes and lymphocytes respond in a similar way in each patient group? Secondly, the more interesting question deals with the mechanistic aspects: Which molecule is responsible for the higher pyroptotic potential of cells from HD?

Undoubtedly, one main difference between BP and HD patients is the huge amount of uremic toxins accumulating in HD. A prominent member of protein-bound uremic solutes is indoxyl sulfate (IS). There are two reports dealing with the effects of IS on NLRP3 activation in the macrophage-like THP-1 model system [8,9]. Unfortunately, both groups showed contradictory results. While Matsuo and colleagues demonstrated IS-triggered NLRP3 activation via the production of oxidative stress, Wakamatsu et al. reported that IS stimulation does not involve caspase-1 activation. Considering these data, we evaluated the IS effects in undifferentiated THP-1 cells (monocyte-like) and differentiated THP-1 cells (macrophage-like). Both model systems have the potential to elucidate the above-mentioned discrepancy, as pyroptosis, in contrast to IL-1β release, can be initiated in the monocyte-like THP-1, whereas macrophage-like THP-1 cells are able to produce IL-1β upon NLRP3 inflammasome specific stimulation.

## 2. Results

### 2.1. Pyroptosis: A Common Feature of Immune Cells of HD Patients

**Figure 1:**
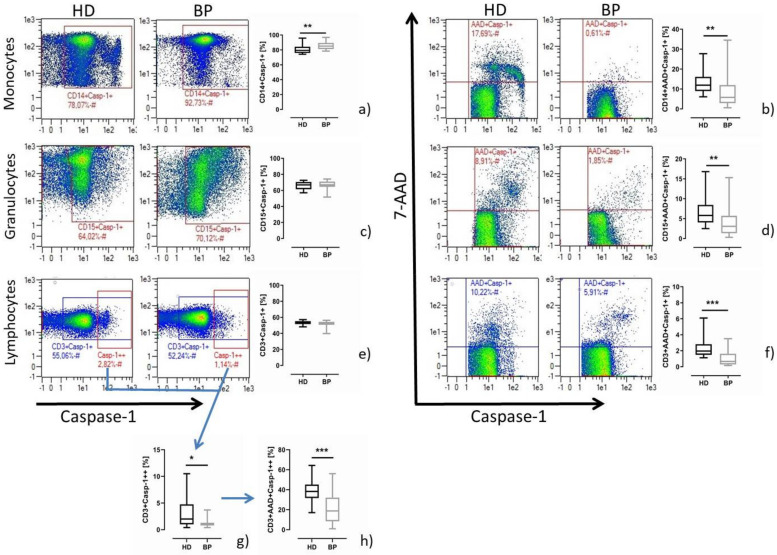
Caspase-1 activity and pyroptosis in leucocyte subsets of HD and BP patients under basal conditions. On the left hand side, representative dot blots (pre-gated by population-specific markers (monocytes, CD14; granulocytes, CD15; lymphocytes CD3)) of a matched pair and the resulting box plot statistics are presented. The data show the frequency of caspase-1 (Casp-1) positive staining monocytes (**a**), granulocytes (**c**) and lymphocytes (**e**) of haemodialysis (HD) and hypertensive patients with healthy kidneys (BP). Lymphocytes staining highly positive for caspase-1 are shown in the Casp-1++ gate (**e**). On the right hand side, pyroptotic results are shown: representative dot blots and resulting box plot statistics are presented. The data show the frequency of pyroptotic (AAD+Casp-1+) monocytes (**b**), granulocytes (**d**) and lymphocytes (**f**) for HD and BP. (**g**) depicts the frequency of highly caspase-1-positive CD3 lymphocytes, among which the frequency of pyroptotic cells (CD3+AAD+Casp-1++) is presented in (**h**). The results are presented as box plots comprising median, 25th and 75th percentile. Statistical differences were analysed by unpaired *t*-test, * *p* < 0.05, ** *p* < 0.01, *** *p* < 0.001.

Immune cells express caspase-1. While in monocytes and granulocytes caspase-1 activity is part of immune defence mechanisms, in lymphocytes caspase-1 activation is thought to be linked to T cell differentiation processes. The frequency of immune cells staining positive for caspase-1 is highest in monocytes, followed by granulocytes and lymphocytes (Figure 1, left hand side). Interestingly, monocytic caspase-1 activity was higher in BP than HD patients, a result that does not apply to granulocytes and lymphocytes (Figure 1, left part). Analysing the pyroptotic events among the caspase-1-positive cells by combined 7-AAD and caspase-1 staining, we find unanimous results demonstrating significantly higher rates of pyroptosis in all immune cells of HD patients (Figure 1, right panel). It is of note that there are subpopulations that stain highly caspase-1-positive (Casp-1++) (Figure 1a,e). However, contrary to lymphocytes and granulocytes, it turned out that in monocytes Casp-1++ is generally more difficult to discern from the caspase-1 low expressing counterparts. At the moment, the characteristic of such a monocyte population remains unclear. However, regarding the caspase-1 positivity of granulocytes and lymphocytes, it is clear that Casp-1++ cells are elevated in HD compared to BP (% granulocytes: 6.4 ± 5.9 vs. 3.4 ± 3.4, *p* = 0.054; % lymphocytes: 2.9 ± 2.7 vs. 1.2 ± 0.7, *p* = 0.011; Figure 1g) and prone to higher pyroptosis (% granulocytes: 35.8 ± 16.2 vs. 22.5 ± 14.2, *p* = 0.008; % lymphocytes: 32.9 ± 12.8 vs. 21.1 ± 16.5, *p* = 0.001; Figure 1h). Further on, to obtain an impression about the priority of pyroptosis in patients versus healthy controls, we recruited three healthy persons, isolated PBMCs and measured pyroptosis. As expected, the monocytic pyroptosis rates were lowest in healthy controls (% pyroptosis; CO: 3.5 ± 1.7 vs. BP: 8.6 ± 8.3 vs. HD: 13.6 ± 6.0).

### 2.2. Pyroptosis in Patients after In Vitro Stimulation with LPS/Nigericin

**Figure 2:**
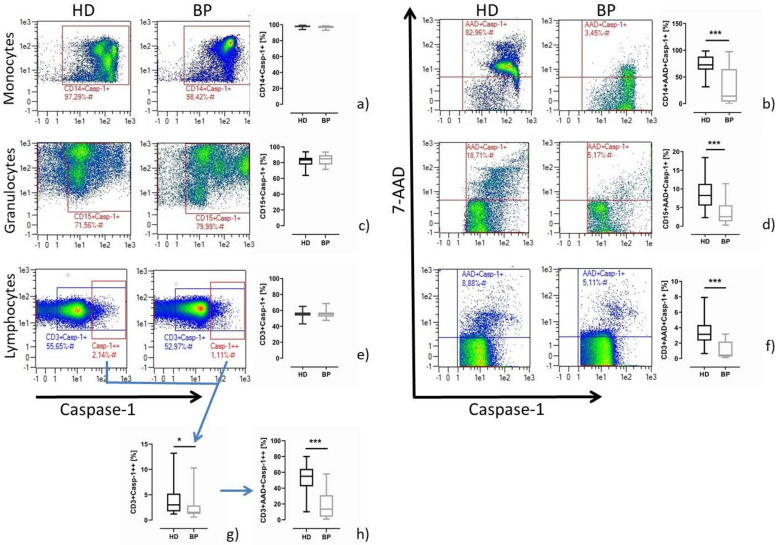
Caspase-1 activity and pyroptosis in leucocyte subsets of HD and BP patients upon 3h45min LPS/15 min nigericin stimulation. On the left hand side, representative dot blots (pre-gated by population-specific markers (monocytes, CD14; granulocytes, CD15; lymphocytes CD3)) of a matched pair and the resulting box plot statistics are presented. The data show the frequency of caspase-1 (Casp-1) positive staining monocytes (**a**), granulocytes (**c**) and lymphocytes (**e**) of haemodialysis (HD) and hypertensive patients with healthy kidneys (BP). Lymphocytes staining highly caspase-1-positive are shown in the Casp-1++ gate (**e**). On the right hand side, representative dot blots and resulting box plot statistics are presented. The data show the frequency of pyroptotic (AAD+Casp-1+) monocytes (**b**), granulocytes (**d**) and lymphocytes (**f**) for HD and BP. (**g**) depicts the frequency of highly caspase-1-positive CD3 lymphocytes, among which the frequency of pyroptotic cells (CD3+AAD+Casp-1++) is presented in (**h**). The results are presented as box plots comprising median, 25th and 75th percentile. Statistical differences were analysed by unpaired *t*-test, * *p* < 0.05, *** *p* < 0.001.

Challenging the isolated cells (PBMCs, granulocytes) with NLRP3 inflammasome-specific stimuli resulted in comparable frequencies of cells staining positive for caspase-1 in HD and BP patients in all three different groups of immune cells (Figure 2, left panel). However, similarly to basal conditions, pyroptosis was significantly enhanced in stimulated immune cells of HD in comparison to BP patients (Figure 2, right panel).

It is of note that the stimulation index (relation of stimulated to unstimulated samples) was quite different among the three immune cell populations. While monocytes of HD patients responded to the LPS/nigericin stimulation by a 6.3 ± 2.9-fold higher pyroptotic rate, the corresponding ratio for BP was 3.6 ± 3.3, *p* = 0.009. Lower stimulation ratios were found for granulocytes (HD vs. BP: 1.5 ± 0.9 vs. 1.1 ± 0.6, *p* = 0.132) and lymphocytes (HD vs. BP: 1.6 ± 0.9 vs. 1.1 ± 0.9, *p* = 0.019). As measured in unstimulated samples, there exists a clearly defined population staining highly positive for caspase-1 (Casp-1++); this is true for lymphocytes and granulocytes, but not for monocytes. The frequency of Casp-1++ cells is not significantly different between HD and BP (% granulocytes; 9.6 ± 6.9 vs. 10.3 ± 5.6, *p* = 0.713; % lymphocytes, 4.0 ± 3.3 vs. 2.3 ± 2.1, *p* = 0.066; Figure 2g). In contrast, pyroptosis rates among Casp-1++ were significantly increased in HD compared to BP patients (% granulocyte: 32.2 ± 18.6 vs. 14.4 ± 11.6, *p* = 0.002; % lymphocytes: 53.4 ± 15.0 vs. 19.5 ± 17.8, *p* = 0.001; Figure 2h). In summary, inflammatory cell death was higher in HD compared to BP at the basal level and after NLRP3 inflammasome specific stimulation.

### 2.3. Caspase-1 Activity and T Helper Cell Differentiation

**Figure 3:**
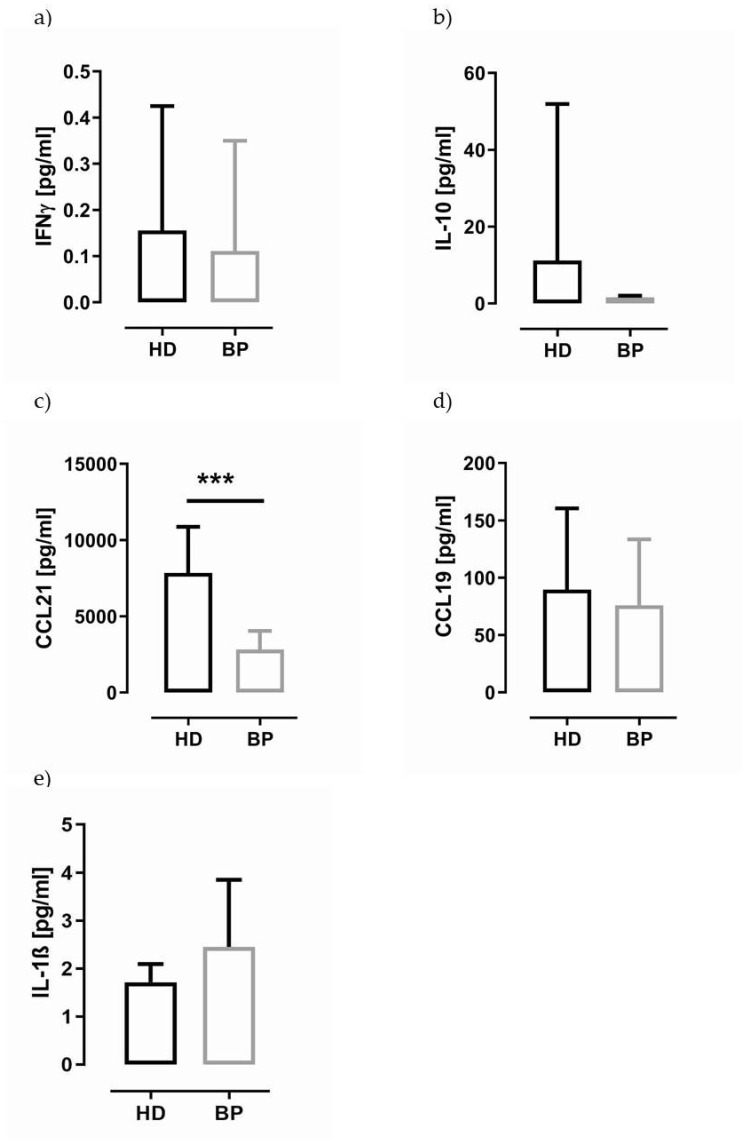
IFN-γ, IL-10, CCL21 and CCL19 concentration in the serum of HD and BP patients. (**a**) depicts interferon-gamma (IFN-γ) serum level; (**b**) interleukin-10 (IL-10) serum concentration; (**c**) CC-chemokine ligand 21 (CCL21), (**d**) CCL19 serum level and (**e**) IL-1 plasma level. Data are presented as columns ± SD. Statistical differences were analysed by unpaired *t*-test, *** *p* < 0.001.

Surprisingly, about 50% of CD3-positive lymphocytes possess caspase-1 activity independently of disease state (Figure 1e and Figure 2e). The caspase-1 expression per lymphocyte (MFI) is significantly higher in HD patients (HD; 9.6 ± 2.7 vs. BP 7.5 ± 1.9, *p* = 0.010). As components of the NLRP3 inflammasome are thought to act in an autocrine manner, thus influencing the T cell differentiation, we flow cytometrically characterised the T cell profile of PBMCs. As demonstrated in Table 1, the T helper cell profile was not significantly different between both groups. In line with these data, we found that IFN-γ serum levels, an indicator of a predominant Th1 response, were not significantly different in both groups (Figure 3a, HD vs. BP, *p* = 0.679).

We also examined the possibility that both patient groups could differ with regard to their T cellular migratory pattern and measured the migratory expression marker CCR7 and its ligands CCL21 and CCL19. CCR7 is highly expressed on naïve and central memory T cells. Further on, it is known that most of the regulatory cells (Tregs) express this molecule. Frequencies of both naïve and central memory CD4 T cells did not differ in both groups. The Treg frequency was significantly elevated in HD patients (Table 1). Correlation analysis, however, could not prove any significant relationship between Treg frequency and corresponding caspase-1 activity (HD: r = −0.106, *p* = 0.718; BP: r = −0.302, *p* = 0.294).

Among both CCR7 ligands, CCL21 is of particular interest in our cohort because it is not only involved in the homing of cells to the endothelial venules in lymph nodes, thus alleviating leucocytic transmembrane trafficking, but it is also a putative marker of cardiovascular disease [10]. In contrast to CCL19, the serum level of CCL21 was significantly increased (Figure 3c,d) in HD. Moreover, IL-10, an anti-inflammatory cytokine, which is also related to Tregs, is slightly elevated in the serum of HD patients (Figure 3b, *p* = 0.089). In line with IL-1β data measured in cell culture, the IL-1β plasma levels were slightly lower in HD patients compared to BP (*p* = 0.065, Figure 3e).

### 2.4. IL-1β in Supernatants of PBMCs and Granulocytes of HD and BP Patients

The basal level (unstimulated PBMCs (uP), unstimulated granulocytes (uG)) of IL-1β protein expression was significantly lower in HD compared to BP, while ex vivo stimulation of PBMCs (sP) and granulocytes (sG) with LPS and nigericin did not result in significantly different IL-1β concentrations (Table 2). It is of note that IL-1β levels in cell culture supernatants of PBMCs were higher in comparison to IL-1β levels measured in granulocytes (HD, uP vs. uG, *p* = 0.568; BP, uP vs. uG, *p* = 0.05). A large difference in IL-1β secretion between monocytes and granulocytes was determined in stimulated samples (HD: sP vs. sG, *p* < 0.001; BP, sP vs. uG, *p* < 0.001). Given that lymphocytes only produce low amounts of IL-1β, which are used in an autocrine manner, the main producers of IL-1β among peripheral blood cells are monocytes.

The question arises if this indicates inflammatory activation in BP patients. However, the IL-6 levels in the culture supernatants of PBMCs were not different in both groups (HD: 18.2 ± 24.4 vs. BP: 23.5 ± 31.0, *p* = 0.498).

### 2.5. Indoxyl Sulfate Can Induce Pyroptosis

Regarding the high pyroptotic rates in blood cells of HD patients, there is the question of whether uremic toxins can be responsible for this effect. Several uremic toxins seem to be related to inflammasome activation, among which trimethylamine-*N*-oxide [11], uremic acid crystals [12] and the protein-bound uremic toxin indoxyl sulfate (IS) are most prominent. Among the three substances, IS appeared the most interesting to us as this toxin is also related to cardiovascular disease but not associated with gout; patients with gout were excluded from the study.

**Figure 4:**
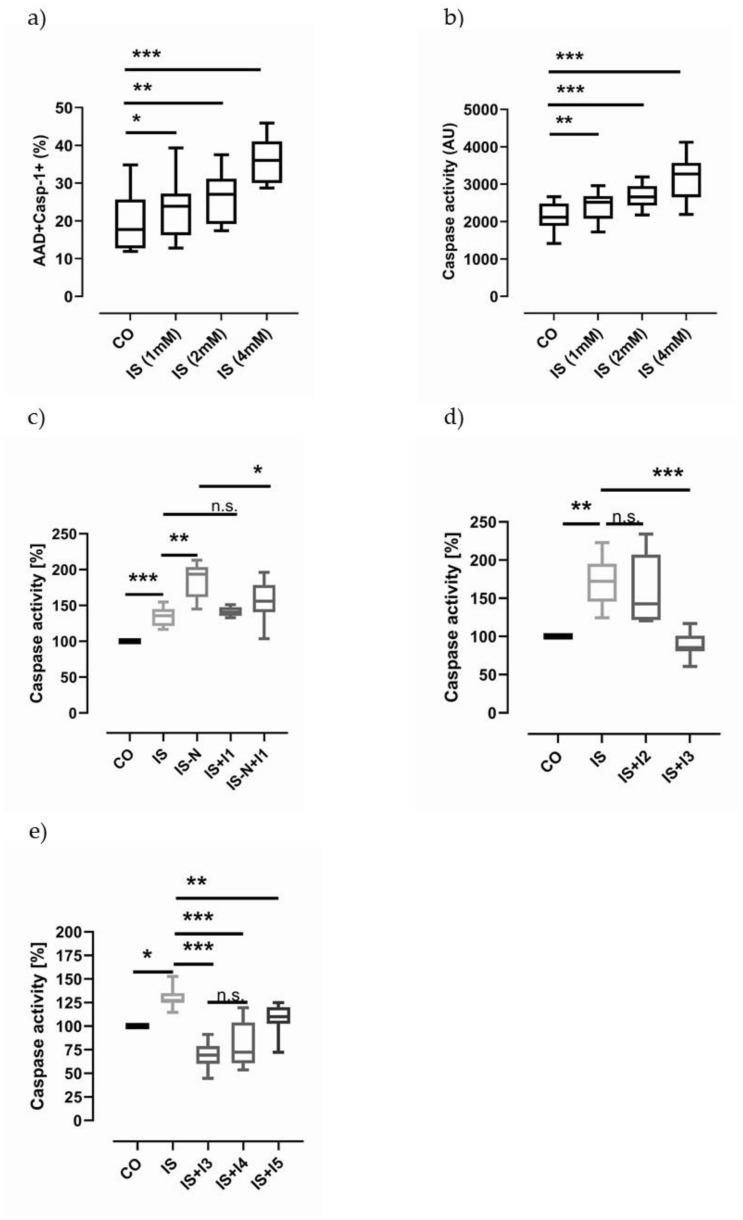
Indoxyl sulfate-dependent caspase-1 activation in monocytic THP-1 cells. (**a**) depicts the indoxyl sulfate (IS) concentration-dependent increase in pyroptosis-positive cells (AAD+Casp-1+, N = 9) in comparison to untreated cells (CO). (**b**) The luminometric Caspase-Glo^®^1 assay confirms that caspase-1 activity is associated with IS levels (N = 6). (**c**) IS (2 mM)-mediated effects can be increased by nigericin stimulation (IS-N). The caspase-1 activity cannot be blocked by treatment of cells with the inhibitor Ac-YVAD-CHO (1µM, IS+I1, N = 8). In contrast, nigericin-derived (N) caspase-1 activity can be inhibited by I1 treatment (IS-N+I1). (**d**) High concentration of Ac-YVAD-CMK (I2, 100 µM) can partially inhibit IS-derived (4 mM) caspase-1 activity (IS+I2), while the pan-caspase inhibitor Z-VAD-FMK (I3, 2.5 µM) totally blocks the induced caspase-1 activity (IS+I3, N = 8). (**e**) IS-derived caspase activity is completely inhibited by the pan-caspase inhibitor (I3, 2 µM) and the inhibitor of caspase-4 and -5 (I4, 2 µM AC-LEVD-CHO), but not by the caspase-8 inhibitor (I5, 2 µM Ac-IETD-CHO; N = 8). The results are presented as box plots comprising median, 25th and 75th percentile. Statistical differences were analysed by one-way ANOVA, using Dunnett’s or Dunn’s multiple comparisons test as post-test, * *p* < 0.05, ** *p* < 0.01, *** *p* < 0.001, n.s.: not significant. Legend: IS (indoxyl sulfate); N: nigericin (21 µM); I1: caspase-1 inhibitor (Ac-YVAD-CHO, 1 µM); I2: caspase-1 inhibitor (Ac-YVAD-CMK, 100 µM); I3: pan-caspase inhibitor (Z-VAD-FMK, 2.5 µM or 2.0 µM, as indicated); I4: caspase-4 (-5) inhibitor (AC-LEVD-CHO, 2 µM); I5: caspase-8 inhibitor (Ac-IETD-CHO, 2 µM).

THP-1 cells are often used as a model system to study the NLRP3 inflammasome-dependent mechanisms. Using a stable monocytic cell line, we take advantage of an easy to handle model system, which is subjected to fewer fluctuations in comparison to isolated PBMCs. IS concentrations in the range of 1 to 4 mM were used to reveal putative mechanistic effects mediated by IS. Flow cytometric analysis demonstrates that pyroptosis is already increased by the stimulation of THP-1 cells at concentrations of 1mM IS (Figure 4a). These data were confirmed by a second luminometric assay, which is based on a different caspase-1-specific substrate compared to the flow cytometric assay (Ac-WEHD-aminoluciferin vs. FAM-YVAD-FMK) (Figure 4b). Further, the caspase-1 activity could be enhanced by supplementing IS-stimulated (2 mM) samples with the NLRP3 inflammasome activator nigericin (Figure 4c). To examine the specificity of the tested caspase-1 substrate, inhibitor probes are used at concentrations reaching from 1–100 µM. Supplementing cells with a low concentration of the caspase-1 specific inhibitor AC-YVAD-CHO (I1, 1 µM), we failed to inhibit IS-mediated effects, whereas the nigericin-driven effect could be blocked (Figure 4c). Challenging IS-stimulated (4 mM) THP-1 cells by very high concentrations (100 µM) of the caspase-1-specific inhibitor Ac-YVAD-CMK (I2), caspase-1 activity was partially blocked (IS: 170 ± 63 vs. IS+AC-YVAD: 128 ± 53), while the incubation of IS-stimulated cells with the pan-caspase inhibitor Z-VAD-FMK (I3, 10 µM) led to a complete inhibition of caspase-1 activity (Figure 4d). To reveal which caspase is affected by Z-VAD-FMK inhibition, we investigated the effects of the inhibitory substrates Ac-LEVD-CHO, which specifically blocks caspase-4 and -5 activities, and Ac-IETD-CHO, an inhibitor substrate of caspase-8. As depicted in Figure 4e, the inhibitor of caspase-4 and -5 totally decreased IS-derived caspase activity, while the inhibitor of caspase-8 was less effective in our experimental setting.

### 2.6. IS Induces the Transcription of Caspase-1, Caspase-4 and IL-1β in Differentiated THP-1 Cells

**Figure 5:**
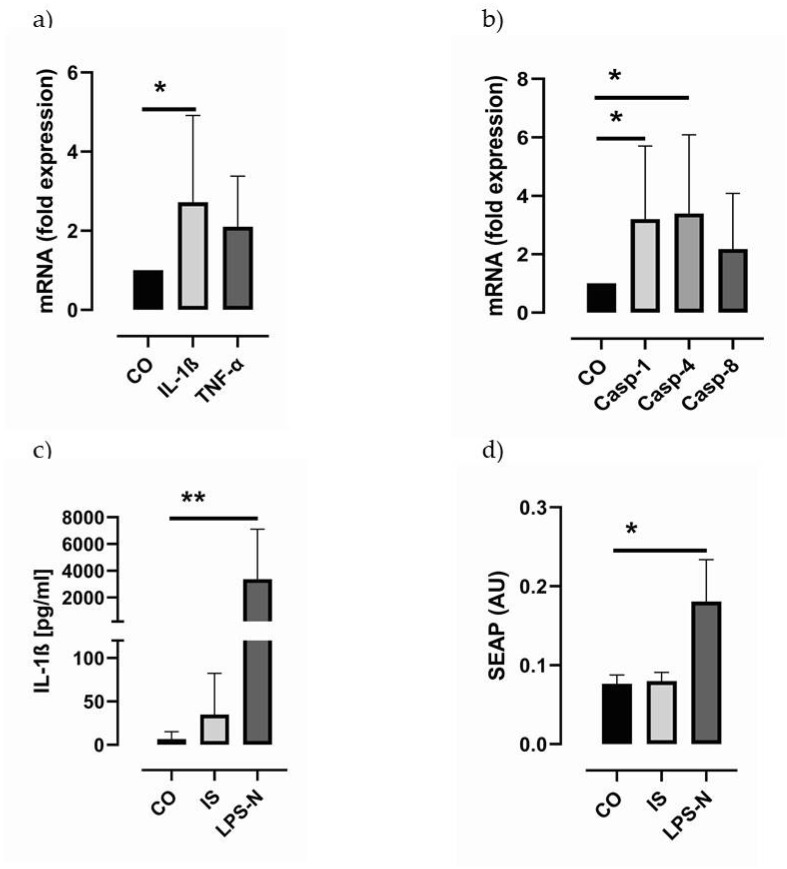
Induction of caspase and IL-1β expression by IS in differentiated THP-1 cells by 4mM indoxyl sulfate. (**a**) depicts transcripts of caspase-1, caspase-4 and caspase-8 (N = 6), (**b**) transcripts of IL-1β and TNF-α (N = 6). (**c**) IL-1β ELISA analysis in THP-1 cells stimulated with 4mM indoxyl sulfate (IS) or LPS and nigericin (LPS-N, N = 6). (**d**) IL-1β-induced SEAP activity (secreted embryonic alkaline phosphatase) in HEK-Blue™ IL-1β cells stimulated with 4mM IS or LPS and nigericin (N = 6). Data are presented as columns ± SD. Statistical differences were analysed by one-way ANOVA using Dunnett’s or Dunn’s multiple comparisons test as post-test, * *p* < 0.05, ** *p* < 0.01.

It is known that IS has a stimulating impact on the mRNA expression of pro-IL-1β in macrophage-like THP-1 cells. We confirmed these data showing a two-fold increase in IL-1β mRNA expression when differentiated THP-1 cells were stimulated with 4 mM IS for 24 h (Figure 5a). There was also a trend for higher TNF-α transcripts upon IS stimulation (Figure 5a). Further, we found a significant increase in caspase-1 and caspase-4 but not caspase-8 mRNA expression in these cells (Figure 5b). However, IL-1β protein levels seem to be only marginally elevated in IS-stimulated samples (Figure 5c). Analysing the biological activity of the THP-1-derived supernatants in the HEK-Blue™ IL-1β cell line, which produces SEAP (secreted embryonic alkaline phosphatase) upon induction with active IL-β, we did not find a SEAP increase in samples stimulated with the IS-derived THP-1 supernatants (Figure 5d). These data support the conclusion that IS does not activate the NLRP3 inflammasome.

## 3. Discussion

In this study, we extended our analysis of pyroptotic events in cells from HD patients. Previously, we demonstrated that among two cardiovascular risk groups, hypertensive versus hypertensive patients with end-stage chronic kidney disease, HD is a prominent factor driving pyroptosis in monocytes [13]. Now, we show that pyroptotic events are not restricted to monocytes but more likely a common phenomenon among immune cells. In accordance with data analysed in monocytes, granulocytes and lymphocytes of HD show elevated pyroptotic rates compared to BP. However, while caspase-1 activity of monocytes and granulocytes is part of a central immune defence mechanism, other NLRP3 inflammasome-related effects are discussed for lymphocytes. All key components of the NLRP3 inflammasome are expressed in T-lymphocytes [14], taking part in T helper cell differentiation. On the one hand, Bruchard et al. demonstrated that the sensor protein NLRP3 fulfils a task as a transcriptional regulator of Th2 differentiation [15]. On the other hand, Arbore and co-workers lend support to the view that complement-driven NLRP3 inflammasome activity delivers autocrine IL-1β for optimal IFN-γ expression by T cells [16]. With regard to our data, we could not provide any evidence that elevated caspase-1 activity in HD is linked to a Th1- or to a Th2-related T cell phenotype. However, HD patients have a 1.6-fold higher frequency of Tregs compared to BP and a 2.6-fold increase in CCL21, a CCR7 ligand that is known to be related to vascular inflammation and heart failure [10]. Taking into account that usually a majority of both naïve and effector/memory Treg subsets express CCR7 [17], there might be the indication for a role of Tregs in endothelial venules of lymph nodes and in endothelial cells of lymphoid tissues, both places of CCL21 production [18]. However, this, of course, is rather speculative than proven. Further, as concluded from correlation analysis, the elevated frequency of Tregs in HD is not related to caspase-1 activity. A different mechanism has to be responsible for that phenomenon.

Beyond doubt, inflammation is a driver of morbidity in HD patients and it is well established that uremic solutes contribute to this phenomenon [19,20]. Among the plethora of uremic toxins, the protein-bound indoxyl sulfate plays a prominent role in cardiovascular and renal disease progression [21,22,23]. IS is derived from tryptophan, which is metabolised by intestinal bacteria into indoles, which are resorbed and then converted to indoxyl sulfate in the liver. The highest concentration of IS ever found in HD was about 1 mM [24]. Our in vitro data show that monocytic THP-1 cells respond to 1 mM IS in a pyroptotic way. It is in agreement with the literature that the activation of the NLRP3 inflammasome in THP-1 cells, likewise in native monocytes, only needs one signal for activation [25]. The pyroptotic response of THP-1 can be enhanced with increasing doses of IS, and the combination of 2 mM IS together with the classical NLRP3 inflammasome stimulus nigericin leads to higher pyroptosis rates compared to the stimulation of THP-1 with 4 mM IS. Therefore, one can conclude that IS is not an archetypical NLRP3 inflammasome activator, but may contribute to its activation. The literature reflects this uncertainty for it is not quite clear whether caspase-1 is activated by IS or not. Matsuo and colleagues reported that IS activates this inflammasome via reactive oxygen species, whereas Wakamatsu et al. did not find a relationship between caspase-1 expression and IS stimulation [8,9]. Therefore, we decided to have a closer look at caspase-1 activation by IS. For such studies, the kind of caspase assay is of fundamental importance. The commonly used assays include Western blot and ELISA, but both assays do not necessarily determine active caspase-1. Fluorometric and luminometric assays measuring the binding or cleavage of caspase-1-specific substrates appear to be more suited to address IS-dependent caspase-1 activation, although some tetrapeptide caspase substrates show overlapping caspase affinities. Interestingly, the greatest caspase-1 catalytic efficiencies (k_cat_/K_M_) were determined for the substrate WEHD [26], but this substrate is also a target of caspases-4 and -5 [27]. The substrate YVAD appears to be more specific for caspase-1, with lesser cross-reactivity compared to WEHD [28]. Thus, the specificity of the caspase-driven effects in the Caspase-Glo assays (WEHD-based) has to be studied in more detail. This can be performed by using specific caspase substrate inhibitors. The specific caspase inhibitor substrates Ac-YVAD-CMK and AC-YVAD-CHO show their biological activity at a concentration range between 1 and 30 µM (according to manufacturers), but in some publications caspase inhibitor concentrations up to 100 µM are used [29]. We failed to inhibit caspase-1 activity using 1 µM of Ac-YVAD-CMK, and even the use of 100 µM of the inhibitor only partially decreased the caspase activity exerted by 4 mM IS. In contrast, the pan-caspase inhibitor Z-VAD-FMK (inhibits the proinflammatory caspases -1, -4 and -5, as well as the apoptotic caspases-2, -3, -6 and -12) totally inhibited the stimulatory effect exerted by 4mM IS. Specifying the pan-caspase inhibition effects by using inhibitory substrates for caspase-4 and -5 (there is no specific inhibitory substrate for both), we clearly found that the main IS-derived effect is dependent on the inflammatory caspases-4 and -5 and to a minor extent on the apoptotic caspase-8 and on caspase-1, respectively.

These results, of course, challenge the data in the ex vivo study [13], and although the authors used the putatively more specific caspase-1 substrate, there remains uncertainty about to which extent the measured pyroptotic rate in monocytes of HD is dependent on caspase-1 and/or caspase-4/-5 activity. Taking mRNA analysis of caspase-4 and caspase-8 into account, we excluded in a recent study that the high pyroptotic events in our HD patients could also result from caspase-4 or caspase-8 action but rather refer to caspase-1 activity [13]. However, mRNA expression analysis may not sufficiently describe the nature of caspase activities. In the light of our new data, caspase activity measurement in combination with substrate inhibition may the best way to answer caspase-related questions.

With regard to the effects exerted by IS, two activating pathways were recently discussed. First, Nakano et al. impressively demonstrated that the organic anion transporter OATP2B1 mediates the uptake of IS into macrophages, and signalling via the DII4-NOTCH pathway and NF-κB activation results in the transcription of proinflammatory genes such as IL-1β, TNF-α and MCP1 [30]. Second, the study of Wakamatsu and colleagues highlighted that the aryl hydrocarbon receptor (AhR), a ligand-activated transcription factor, is not only a receptor for dioxins but also for tryptophan-derived uremic toxins such as IS [9]. The activation of AhR by IS is also a booster of NF-κB activation and increased TNF-α-induced leucocyte recruitment to endothelial cells in the vascular wall [31], while pro-IL-1β was enhanced thereof in macrophages [9]. Last but not least, the AhR pathway does also affect lymphoid cells as AhR activation leads to the generation of Tregs [32,33]. Thus, it is possible that high pyroptotic rates of immune cells, elevated Tregs and increased levels of the chemokine ligand CC21 in HD patients are a further mosaic piece representing the enhanced inflammatory disorder in these patients.

IS appears to be associated with many negative clinical outcomes in HD [34]; however, the removal of indoxyl sulfate by dialysis in clinical studies did not prove any benefit [35,36]. One could also think about preventing excessive cell death in HD patients by the inhibition of different inflammatory caspases. However, as far as we know—although many caspase inhibitors are patented so far, none have been tested in clinical studies—the toxicity and poor pharmacokinetic profile of these substances prevented their use [37]. New caspase inhibitors with lower toxicity profiles have to be developed.

However, there are limitations of this study. First, the study is small, and second, we only analysed pyroptotic effects of monocytes and lymphocytes in the context of isolated PBMCs. Third, we tried to overcome the lack of mechanistic caspase-4-dependent assays in PBMCs by studying effects in the THP-1 model using high concentrations of indoxyl sulfate (1–4 mM). The results derived from the model system, however, appear to offer an explanation as to how IS could contribute to the increased pyroptotic effects measured in HD in comparison to hypertensive BP patients.

## 4. Conclusions

We demonstrated in this study that pyroptosis affects all immune cells and the level of pyroptosis is higher in HD patients compared to another cardiovascular risk population, namely hypertensive patients with intact kidney function. Analysing the effect of indoxyl sulfate in the THP-1 model systems, we provide evidence for the first time that the inflammatory caspase-4 and/or caspase-5 can produce pyroptosis in monocyte-like cells without increasing IL-1β levels. New studies must prove if proinflammatory caspase-4/-5 is a driver of pyroptotic death in HD, while caspase-1 activity is elevated in hypertensive patients with intact kidney function.

## 5. Materials and Methods

### 5.1. Study Population

The study population and the corresponding clinical procedures were described in [13]. In short, the observational study enrolled 20 end-stage renal failure patients (HD) who were on maintenance haemodialysis for > 6 months and 20 hypertensive patients (BP) with intact kidney function. HD patients were recruited from the outpatient unit of the Department of Internal Medicine II, and patients with intact kidney functions came from the nephrological and cardiological units of the hospital of the Martin Luther University Halle-Wittenberg. Patients were >18 years of age and those patients with active malignancy, active infections (CRP > 50 mg/L), systemic autoimmune disorders (systemic lupus erythematosus, granulomatous polyangiitis) and neurologic disorders were excluded. Further on, HD patients suffering from gout disease—a well-known activator of NLRP3 inflammasome—were also not included in the study. To prevent bias in the comparison of both groups, patients with intact kidney function were age-, gender- and diabetes-matched to HD patients. For the classification of the disease-specific NLRP3 activation pattern, we recruited apparently healthy subjects (2 m/1f; 50.7 ± 11.0 years, 24.7 ± 2.3 BMI (kg/m^2^)).

### 5.2. PBMC/Granulocyte Isolation

PBMC isolation was described in [10]. Granulocytes were isolated from erythro-sediments of ficollized PBMCs by hypertonic solution. About 10 × 10^6^ of each PBMC preparation was stored in FBS/10% DMSO (Merck-Millipore, Darmstadt; Roth, Karlsruhe, Germany) at −80 °C. The T-helper cell profile was analysed from these samples.

### 5.3. NLRP3 Inflammasome Stimulation Model

The NLRP3 inflammasome model was described in [10].

### 5.4. Antibodies for Flow Cytometry

The following antibodies were used for analysis of PBMCs in the caspase-1 assay: Anti-CD16 APC (clone 3G8, BD Biosciences, Heidelberg, Germany), -CD14 PeCy7 (clone 61D3, ThermoFisher Scientific, Darmstadt, Germany), -CD15 eFluor450 (clone HI98, ThermoFisher Scientific, Darmstadt, Germany), -CD3 VB (clone BW264/56, Miltenyi Biotec, Bergisch-Gladbach, Germany), 7-AAD (ThermoFisher Scientific, Darmstadt, Germany). The T cell profile was characterised by staining with: 7-AAD, anti-CCR6 PE, -CD25 PeCy7, -CD127 APC, CD45RO FITC (all ThermoFisher Scientific, Darmstadt, Germany), -CD8 PE, -CCR7 PeCy7, CXCR3 AF700 (all BD Biosciences, Heidelberg, Germany), -CD3 VB and -CD4 FITC or APC (all Miltenyi Biotec, Bergisch-Gladbach, Germany Bergisch-Gladbach, Germany). Samples were analysed on the MACS Quant analyser (Miltenyi Biotec, Bergisch-Gladbach, Germany) using MACS Quantify software. Gates were set according to FMO (fluorescence minus one) controls.

### 5.5. Determination of Active Caspase-1 by Flow Cytometry

Caspase-1 was flow cytometrically detected by the FAM-FLICA^®^ Caspase Assay using the FLICA^®^ probe (FAM-YVAD-FMK) as described by the manufacturer (BioRad, Feldkirchen, Germany). The fluorescent inhibitor binds to activated caspase-1. One hour before ending the regular incubation period, cells (0.25 × 10^6^ cells, PBMCs or granulocytes) were pelleted and re-suspended in sterile PBS/0.5% HSA containing the FLICA^®^ probe. The incubation was continued for 1 h. Samples without FAM-YVAD-FMK were used as negative controls. Cells were counterstained with population-specific markers (PBMCs: CD14 PeCy7, CD3 VB, CD16 APC; granulocytes: CD15 eFluor450, CD14 PeCy7, CD3 VB. 7-AAD) (ThermoFisher Scientific). Staining in combination with caspase-1 positivity was applied for detection of pyroptosis (AAD+Casp-1+).

### 5.6. THP-1 Cell Culture

THP-1 cells were regularly maintained in RPMI1640 culture medium containing 10% FCS, 2 mM glutamine, 1% penicillin and streptomycin. For experiments with indoxyl sulfate, cells were cultured in RPMI medium containing 4% human serum albumin (Behring, Marburg, Germany) instead of FBS. Cells were stimulated as indicated with different concentrations of indoxyl sulfate (Sigma-Aldrich) for 24h. In some cases, nigericin was added (21 µM) for the last hour of the incubation period. For differentiation experiments, 1 × 10^6^ THP-1 cells were incubated in 6-well plates (VWR International GmbH, Ismaning, Germany) in the presence of phorbol 12-myrestate-13-acetate (PMA, 100 ng/mL; Sigma-Aldrich) for 72h, followed as indicated by specific stimulation.

### 5.7. Determination of Active Caspase-1 by Caspase-Glo^®^1 Inflammasome Assay

The Caspase-Glo^®^1 Inflammasome Assay was purchased from Promega, Heidelberg, Germany. A total of 50,000 THP-1 cells each were seeded in quadruplicates in a white, opaque-walled 96-well plate (Greiner, Frickenhausen, Germany). Cells were incubated for 24 h with or without indoxyl sulfate (concentration as indicated in the text) and caspase-1 inhibitors (Ac-YVAD-CHO (1 µM, Promega), Ac-YVAD-CMK (100 µM)) or the pan-caspase inhibitor Z-VAD-FMK (10 µM or 2.0 µM, as indicated) (both InvivoGen, Toulouse, France), the caspase-4/-5 inhibitor (Ac-LEVD-CHO, 2 µM) and the caspase-8 inhibitor (Ac-IETD-CHO, 2 µM) (both Merck Millipore, Darmstadt, Germany), respectively. Samples supplemented with nigericin (21 µM) incubated for the last 60min of the assay served as positive controls and media without cells as negative controls. Caspase-1 activity was determined after 1h by luminometry using the Infinite M200 Pro analyser (Tecan, Crailsheim, Germany).

### 5.8. RNA/cDNA/qPCR

RNA was isolated from THP-1 cells by Direct-zol™ RNA MiniPrep Plus Kit (ZymoResearch).

The RNA concentration and quality (260/280 ratio: 1.8 ± 0.2) were tested by Nanodrop technique (PEQLAB Biotechnologie GmbH, Erlangen, Germany). Equal amounts of RNA (100 ng) were reverse transcribed using the FastGene Scriptase Basic cDNA Kit (Nippon, Düren, Germany).

Caspase-1 (Hs00354836_m1), caspase-4 (Hs01031951_m1), caspase-8 (Hs01018151_m1), IL-1β (Hs00174097_m1), TNF-α (Hs00985639_m1) and the house-keeping gene RPL37a (Hs01102345_m1) mRNA expressions were analysed using TaqMan probes (ThermoFisher Scientific) and the qPCRBIO Probe Mix High-ROX (Nippon). The samples were processed in duplicate on a StepOnePlus Cycler (ThermoFisher Scientific). Data were normalised to the house-keeping gene, related to medium controls and expressed as x-fold difference (2^−ddCt^ method).

### 5.9. IL-1β Determination Using HEK-Blue™ IL-1β Indicator Cells

HEK-Blue™ IL-1β cells, antibiotics and Quanti-blue™ solution were purchased from InvivoGen. The assay was performed according the instruction of the manufacturer. In short, HEK-Blue™ IL-1β cells expressing the IL-1 receptor complex are sensitive to IL-1β. Stimulation of cells with IL-1β leads to the induction of SEAP (secreted embryonic alkaline phosphatase), which is able to process the Quanti-Blue™ substrate. The SEAP levels can be measured spectrophotometrically. The cells were regularly grown in DMEM with high glucose (Sigma-Aldrich) supplemented with 2 mM glutamine, 10% FBS, 100 U/mL penicillin, 100 µg/mL streptomycin and 100 µg/mL normocin. For assay conditions, 50 µL cell suspension (45,000 cells) in medium without normocin was seeded in 96-well plates (VWR). Each well was supplemented with 150 µL supernatant harvested from THP-1 cells. After a 24h incubation period (5% CO_2_, 37 °C), 20 µL of each supernatant was transferred to a 96-well plate containing 180 µL Quanti-blue™ solution per well. After 3h of incubation at 37 °C, the samples were measured on a EL_x_ 808 ELISA reader (BioTek Instruments Corp., Berlin, Germany) at 630–650 nm.

### 5.10. Cytokine Analysis

IFN-γ was determined from serum samples by ELISA technology (IBL International, Hamburg, Germany). IL-10 serum and IL-1β plasma levels were flow cytometrically analysed using BD™ Cytometric Bead Array (CBA, BD Biosciences). THP-1 and PBMC culture supernatants were screened for IL-1β und IL-6 using the IL-1β and IL-6 ELISA Kits (R&D Systems, Wiesbaden, Germany and IBL). CCL19 and CCL21 ELISA Kits were purchased from Sigma-Aldrich.

### 5.11. Statistics

Results are expressed as mean ± SD. All continuous variables were controlled for normal distribution using the D’Agostino–Pearson omnibus test. Comparisons of data of HD and BP patients were analysed by unpaired *t*-test or Mann–Whitney test. CaspaseGlo^®^1 assays were analysed by one-way ANOVA followed by Dunnett’s or Dunn’s multiple comparisons test as appropriate. All calculations were carried out using the SPSS 21.0 (SPSS Inc., Chicago, USA) or GraphPad Prism 9.2.0 statistics software (GraphPad Software Inc., La Jolla, CA, USA). The level of significance was set at *p* < 0.05.

## Figures and Tables

**Table 1:** T cell characterisation of HD and BP.

	HD	BP	*p*-Value
CD4 (%)	56.5 ± 16.6	57.3 ± 15.3	0.874
CD8 (%)	31.9 ± 18.8	32.1 ± 13.9	0.735
Th1 (CD4+CXCR3+CCR6-)	37.1 ± 18.1	44.8 ± 18.3	0.194
Th2 (CD4+CXCR3-CCR6-)	61.0 ± 18.4	53.5 ± 18.1	0.227
Th17 (CD4+CXCR3-CCR6+)	1.2 ± 0.9	1.1 ± 0.8	0.563
Tregs (CD4+CD25+CD127low)	4.9 ± 2.0	2.6 ± 1.7	00.004
%CD4+CD28+CD45RO-CCR7+ (CD4+CCR7+ naive cells)	24.4 ± 16.7	27.4 ± 16.5	0.650
%CD4+28+45RO+CCR7+ (CD4+ central memory cells)	56.8 ± 18.3	58.4 ± 16.0	0.840

Results are expressed as mean ± SD. The differences in the two groups were analysed by unpaired *t*-test.

**Table 2:** IL-1β in supernatants of PBMCs and granulocytes of HD and BP patients.

	HD	BP	Statistics
uP: IL-1β (pg/mL)	4.6 ± 6.6	17.4 ± 29.7	0.001
sP: IL-1β (pg/mL)	5409 ± 3166	5507 ± 4620	0.678
uG: IL-1β (pg/mL)	2.5 ± 0.6	7.1 ± 5.7	0.001
sG: IL-1β (pg/mL)	275 ± 199	890 ± 1564	0.198

Abbreviations: uP: unstimulated PBMCs; sP: LPS/nigericin-stimulated PBMCs; uG: unstimulated granulocytes; sG: LPS/nigericin-stimulated granulocytes. Results are expressed as mean ± SD. The differences in the two groups were analysed by unpaired *t*-test or Mann–Whitney test.

## Data Availability

Not applicable.
